# Stem Cells and Infertility: A Review of Clinical Applications and Legal Frameworks

**DOI:** 10.3390/jpm14020135

**Published:** 2024-01-24

**Authors:** Gaspare Cucinella, Giuseppe Gullo, Erika Catania, Antonio Perino, Valentina Billone, Susanna Marinelli, Gabriele Napoletano, Simona Zaami

**Affiliations:** 1 IVF Unit, Department of Obstetrics and Gynecology, Villa Sofia Cervello Hospital, University of Palermo, 90146 Palermo, Italy; gaspare.cucinella@unipa.it (G.C.); erika.catania0702@gmail.com (E.C.); antonio.perino@unipa.it (A.P.); valentina.billone@gmail.com (V.B.); 2 School of Law, Marche Polytechnic University, 60121 Ancona, Italy; susanna.marinelli@tiscali.it; 3 Department of Anatomical, Histological, Forensic and Orthopedic Sciences, “Sapienza” University of Rome, 00161 Rome, Italy; gabriele.napoletano@uniroma1.it (G.N.); simona.zaami@uniroma1.it (S.Z.)

**Keywords:** infertility, assisted reproductive technologies (ART), human embryonic stem cells (ESCs), induced pluripotent stem cells (iPSCs), mesenchymal stem cells (MSCs), ovarian stem cells (OSCs), spermatogonial stem cells (SSCs), ethics and legal implications

## Abstract

Infertility is a condition defined by the failure to establish a clinical pregnancy after 12 months of regular, unprotected sexual intercourse or due to an impairment of a person’s capacity to reproduce either as an individual or with their partner. The authors have set out to succinctly investigate, explore, and assess infertility treatments, harnessing the potential of stem cells to effectively and safely treat infertility; in addition, this paper will present the legal and regulatory complexities at the heart of stem cell research, with an overview of the legislative state of affairs in six major European countries. For couples who cannot benefit from assisted reproductive technologies (ART) to treat their infertility, stem-cells-based approaches have been shown to be a highly promising approach. Nonetheless, lingering ethical and immunological uncertainties require more conclusive findings and data before such treatment avenues can become mainstream and be applied on a large scale. The isolation of human embryonic stem cells (ESCs) is ethically controversial, since their collection involves the destruction of human embryonic tissue. Overall, stem cell research has resulted in important new breakthroughs in the treatment of infertility. The effort to untangle the complex web of ethical and legal issues associated with such therapeutic approaches will have to rely on evidence-based, broadly shared standards, guidelines, and best practices to make sure that the procreative rights of patients can be effectively reconciled with the core values at the heart of medical ethics.

## 1. Introduction

Infertility is a condition characterized by the failure to achieve clinical pregnancy after 12 months of regular, unprotected sexual intercourse. Several risk factors are linked to such a condition: a woman’s age, lifestyle (drug use, smoking, alcohol consumption), sexually transmitted diseases, pelvic inflammatory disease, obesity, PCOS, and diabetes. Also, tubal, ovarian, and uterine diseases can contribute to female infertility (endometriosis for example). Finally, infertility may be connected to endocrinological diseases or genetic disorders (Turner syndrome, Klinefelter syndrome, etc.). Infertility has an etiology which is linked to female causes in 40% of cases, and to male ones in 40%, while 10–20% involve both and 10% are idiopathic [1]. Conventional treatments for male infertility include improvement in sperm quality, surgical treatment of varicocele, and administration of gonadotropins or antioxidants [2,3]. As far as female infertility is concerned, there are several possible treatments: gonadotropins; GnRH; FSH; LH, such as ovulation-inducing drugs; clomiphene citrate or letrozole in case of PCOS; bromocriptine or cabergoline to treat hyperprolactinemia. After the administration of these different treatments, chosen in correlation to patient, regular follicular monitoring is necessary with ultrasonography [4,5,6]. Currently, the most widespread assisted reproductive technologies (ART) are intrauterine insemination (IUI), in vitro fertilization (IVF), and intracytoplasmic injection (ICSI). But if a gamete deficiency is proved, because of genetic defects, ART is not the best choice. In such a context, stem cells provide new hope. The aim of this review is to evaluate the use of stem cells and assess their efficacy and safety in infertility treatment.

## 2. Materials and Methods

A broad-ranging search was performed in PubMed/MedLine, Web of Science (WoS), and the Cochrane Database to retrieve studies that analyze the application of stem cells as a therapeutic option for infertility. The search string for the clinical applications of stem cells in infertility treatments included the combination of the key words “stem cells” and “infertility—IVF”; the ethical, legislative, and regulatory research comprised the string “stem cell research ethics”, “legal and regulatory frameworks”, and “stem cell research guidelines and best practices”. All studies were analyzed and selected for their relevance and data quality. Ultimately, 134 sources were included, spanning the 1988–2023 time period. 

## 3. Results

### 3.1. Stem Cells and Female Infertility Conditions

Firstly, it is worth pointing out that stem cells are potentially applicable in a broad array of infertility conditions. Primary ovarian insufficiency (POI), for instance, is a condition of irreversible decline in ovarian function. In addition, long-term low estrogen levels are associated with vasomotor symptoms, urogenital symptoms, osteoporosis, type II diabetes, and cardiovascular and cerebrovascular adverse repercussions. The etiological assessment of POI is highly relevant from a clinical perspective, since various factors can be linked to such a condition [7]. Noteworthy causes are, for instance, follicular reduction, accelerated follicular atresia, and abnormal egg function. Among the most potentially valuable therapeutic avenues for POI, tissue engineering materials combined with mesenchymal stem cells certainly deserve to be mentioned [8]. The use of stem cells and biomaterials has reportedly been confirmed as a viable option for the treatment of POI [9]. Premature ovarian failure (POF) is a relatively widespread reproductive disorder that is linked to premature menopause, higher gonadotropin levels, and estrogen shortages before the age of 40; its etiologies and pathogeneses have not yet been completely clarified [10]. Furthermore, such a syndrome is frequently associated with a host of perimenopausal symptoms such as hot flashes, night sweats, hair loss, skin dryness, mucous membranes, decreased libido, and sleeping and mood disorders. Although hormone replacement therapy (HRT) is currently the chief therapeutic option to treat POF, the use of bone marrow mesenchymal stem cells (BMSCs) has been shown to positively affect ovarian reserve function. Such a beneficial effect is the result of various dynamics and mechanisms such as homing, paracrine, regulation of ovarian angiogenesis, anti-fibrosis, anti-inflammatory, anti-apoptosis, and immune regulation. Conditioned medium derived from BMSCs contains a variety of cytokines, such as vascular endothelial growth factor (VEGF), hepatocyte growth factor (HGF), and insulin-like growth factor (IGF-1) among others. Such cytokines can inhibit apoptosis and favor the proliferation of GCs in vivo or in vitro; hence, they have been reported to play an important role in BMSCs in terms of improving ovarian function. 

### 3.2. Stem Cells Variants and Multiple Applications in Infertility

Stem cells are undifferentiated cells that, if necessary, can self-renew and differentiate. They can repair damaged tissues. Like Saha et al. [11] describe in their paper, there are several kinds of stem cells. Infertility therapeutic options based on stem cells can either rely on direct transplantation of stem cells or their paracrine factors into reproductive organs or on in vitro differentiation into germ cells or gametes. The latter can play a major role through various mechanisms and dynamics: paracrine factors can in fact can trigger differentiation of surrounding cells into mature cell lines; they can bring about the modulation of inflammatory or reparative processes in surrounding tissues; lastly, they can affect the actions of the stem cells (particularly MSCs) which secrete them. Also noteworthy is the ability of such factors to enable one-way conversations between stem cells and more differentiated cells. Animal models have pointed to the ability of such options to improve reproductive outcomes in animal models; however, there are still not enough conclusive data support their successful use in human beings. Table 1 summarizes and succinctly elaborates on the stem cell types used in infertility treatments, their distinctive traits, and current therapeutic applications in reproductive medicine.

Bone-marrow-derived stem cells combined with activated platelet-rich plasma, have been shown to hold promise in terms of their potential to positively impact reproductive outcomes in patients with age-related infertility, further improving the restorative effects of platelet-rich plasma alone [44]. Factors such as the autologous nature of stem cell factors collected by noninvasive mobilization, their combination with platelet-rich plasma, and the local administration route seem to point to stem cells combined with activated platelet-rich plasma treatment as a potentially effective and safe pathway for future clinical application; however, research data on human ovarian samples are still inconclusive. To generate PGCs, precursors of sperm and egg cells, and induce iPSCs, adult stem cells from male and female gonads and pluripotent stem cells such as ESCs were used [45]. In their systematic review, Saha et al. [11] describe the use of stem cells in various disorders such as Asherman Syndrome, a condition characterized by amenorrhea following a uterine cavity injury. The resulting adhesions give rise to infertility, abortion, and chronic pelvic pain [46]. The main cause has been reported to be postpartum endometrial courettage [47]. Several clinical studies have shown improvement of fertility in animal models through bone marrow, menstrual blood, and mesenchymal stem cells [48]. That makes their use in human infertility treatments rather promising. Saha et al. [11] reported another important cause of infertility: premature ovarian insufficiency (POI). Several papers show the efficacy of ovarian stem cells with stimulation of the AKT pathway to improve fertility in this condition [49,50,51]. There is another disorder linked to irregular menstruation, obesity, atypical hair growth, and infertility: polycystic ovarian syndrome [52]. Stem cells could be used to keep PCOS clinical symptoms at bay, suppressing inflammation and producing anti-inflammatory cytokines. Just as noteworthy is the generation of viable oocytes from induced pluripotent stem cells differentiated from male cells, as documented in a 2023 study by Murakami et al. using animal models [16]. Finally, different studies are testing the use of stem cells in endometriosis and azoospermia with promising results. Saha et al. [11] have elaborated on the future prospects for stem cells and infertility. They cite very small embryonic-like stem cells (VSELs) found in human bone marrow [53] with capacity to be differentiated into germinal cells and into different organs cells during embryonic development. They also repair any organ damage [54]. On the other hand, Saha et al. [11] describe micro-RNA (miRNA) and stem-cell-based therapies. miRNA plays an important role in genetic expression of stem cells and in mRNA stability [55]. For example, miR-10 and miR-146a, isolated in stem cells, can improve ovarian function in mice and prevent granulosa cells apoptosis [56]. Certainly, stem cell therapy has progressed to such a degree that further long-term development needs rigorous planning, in addition to strict oversight to guarantee an acceptable degree of safety, accuracy, and quality. Such standards are non-negotiable if stem-cell-based therapeutic avenues are to become ever more mainstream for the potential benefits of countless patients, and are essential even from a legal and ethical standpoint.

### 3.3. Ethics and Legal Implications

Since autologous stem cells are more ethically tenable, safe, and non-immune, the clinical application of such cells has more potential in terms of future therapeutic prospects. On the other hand, ethics and moral implications arising from embryonic stem cells obviously have a lot to do with how the legal and moral status of the embryo is assessed, and whether and to what extent it is deemed worthy of protection. It is therefore quite a different scenario from the one involving induced pluripotent stem cells (iPSCs) and adult stem cells, which are unrelated to embryo status [57]. A discussion centered around ethics and legal assessment standards for such types of stem cells is therefore necessary and should revolve around the possible risks linked to stem cell interventions. Specifically, aspects still in need of clarification have to do with the possible damage which could arise from still under-researched and inadequately validated stem cell procedures, how the informed consent process should be structured for such procedures to be sound from a medicolegal perspective, and finally, the lingering doubts involving ownership and confidentiality of donor information [50]. It is therefore worth briefly elaborating on the various approaches implemented in major European countries when it comes to regulating stem cells research in order to reconcile ethics viability with the needs and innovations of medical scientific research in an area which holds great promise in reproductive medicine and beyond. 

Ethical considerations are of the utmost importance in all medicine, and are eminently relevant in the practice of reproductive medicine, endocrinology, and infertility care. In addition, when treatments relying on stem cells are applied to medically assisted reproduction, the ethics and legal quandaries arising from the latter should be taken into account [58,59,60,61]. It is no wonder that several countries allow for conscience-based refusal from healthcare professionals who feel that such practices conflict with their deeply held moral beliefs [62,63,64], yet it is of the utmost importance that we find viable ways to reconcile such a right with the reproductive rights of couples [65,66]. Such complexities need to be governed by unequivocal standards and criteria that are both evidence-based and as broadly shared as possible at the international level, especially as fast-developing technological advancements seem to outpace our ability to devise tenable, well-balanced, and evidence-based guidelines and best practices to maximize effectiveness while at the same time safeguarding the core values that shape medical ethics [67,68,69,70,71]. Embryonic stem cells are undifferentiated pluripotent cells that can indefinitely grow in vitro. They are derived from the inner mass of early embryos. Because of their ability to differentiate into all three embryonic germ layers, and finally into specialized somatic cell types, human embryonic stem cells certainly constitute a valuable element for research focused on developmental biology and cell replacement therapy. They are usually isolated from excess human IVF embryos [72,73]. Research centered around stem cells and their use in the creation of human embryos is viewed by many as challenging and controversial, if not outright untenable, from the bioethics perspective. The lack of a clean-cut consensus is reflected in the different legislative and regulatory approaches put in place by national lawmakers. Yet, the unavailability and illegality of a therapeutic option in a given country may drive those who seek such treatments, and can afford it, to travel to countries where such practices are legal. That poses an element of access inequality and financial discrimination, as it happens, for instance, with “procreative tourism” [58,59,74]. European countries have codified varying degrees of restrictions affecting the way and extent to which stem cell research can be lawfully undertaken. Table 2 briefly summarizes the legal and regulatory scenarios in six major European countries, selected as meaningful samples in terms of population size.

It is worth remarking that, when stem cells are isolated, embryos are not fully killed: at least one embryonic cell, that is a stem cell, does survive. The life of stem cells cannot be qualified as independent. Nevertheless, the embryo’s life is not completely destroyed and continues in a primitive way of life; hence, there is no outright destruction in the strict sense [92]. In the United States, the 2016 Guidelines for Stem Cell Research and Clinical Translation (ISSCR), updated in 2021 based the prohibition of research on embryoids after 14 days on a “broad international consensus that such experiments lack a compelling scientific rationale, raise substantial ethical concerns and/or are illegal in many jurisdictions” [93]. Nicolas et al. [94] pointed to the need to start a public debate involving all stakeholders, scientists, research policy experts, bioethicists, and community members in order to weigh an extension of the 14-day rule and possibly revise the Dickey–Wicker Amendment which prohibits the United States Department of Health and Human Services (HHS) from using appropriated funds for the creation of human embryos for research purposes or for research in which human embryos are destroyed. In 2001, under the George W. Bush administration, such guidelines were amended, limiting federal funds to only the stem cell lines existing as of 9 August 2001, which was then estimated at approximately 60 cell lines; however, many of those lines eventually proved unusable [95].

## 4. Discussion

One of the main cornerstones of reproductive biology is that women have a finite ovarian reserve, which is set from the very time they are born. This theory has been questioned recently by the discovery of ovarian stem cells which are purported to have the ability to form new oocytes under specific conditions postnatally. Almost a decade after their discovery, ovarian, or oogonial, stem cells (OSCs) have been isolated in mice and humans but remain the subject of much debate. The ideal fertility preservation approach would prevent delays in commencing life-saving treatment and avoid transplanting malignant cells back into a woman after treatment: OSCs can be a viable route to such an end [96]. Based on the recent encouraging results of studies [97] conducted on OTCs, particularly several involving patients with oncological or autoimmune conditions predisposing them to premature ovarian insufficiency and/or infertility, OTC and its subsequent transplantation could be proposed as an alternative to HRT [98,99].

hMensSCs increased the ovarian weight, plasma E2 levels, and follicle numbers in mice [100]. Amniotic fluid stem cells can differentiate into granulosa cells, which inhibit follicular atresia and maintain healthy follicles [101]. Wang et al. [14] found that hESC-derived endometrial cells can support endometrial repair and functional recovery [102]. ESCs were obtained from cloned blastocysts, in turn obtained from somatic cell nuclear transfers (SCNTs) (the resulting embryonic stem cells were called Kitw/Kitwv, ntESCs) [103]. Marinaro et al. [104] demonstrated that extracellular vesicles derived from EnMSCs can elicit an antioxidant effect and be helpful when used as IVF coadjutants. This study relied on endMSCs isolated from human menstrual blood and characterized according to multipotentiality and surface marker expression prior EV-endMSCs isolation. The conclusion was that increased developmental competence of the embryos could be partly mediated by the EV-endMSCs’ ROS scavenger activity [104]. The endometrial side population (ESP) constitutes a mixed population, mostly made up of precursors of endothelial cells [105]. Adequate uterine vascularity and the regulating cells/factors are necessary preconditions at the time of implantation. Inappropriate endometrial angiogenesis and immunity can result in reproductive failure, especially in recurrent miscarriage and recurrent implantation failure (RIF) [106].

Tersoglio et al. have accounted for endometrial changes before and after the transfer of endometrial mesenchymal stem cells (enMSCs) in a population of women with thinned endometria, with absence or hypo-responsiveness to estrogen and RIF; a substantially high level of increase in endometrial thickness was ultimately reported following the inoculation of enMSCs, pointing to the considerable regenerative potential of such an approach [107]. MSCs are plentiful and substantially capable of self-renewal differentiation. Another considerable advantage is that they are more ethically sustainable and can be applied as a viable therapeutic avenue for female infertility, potentially offering an alternative to intrauterine insemination, in vitro fertilization, drug-based treatments, and surgical procedures [22].

Although it is not yet a well-established technology, oocyte cryopreservation has been getting increasingly widespread in assisted reproductive technologies in response to the growing demands of patients’ sociological and pathological conditions. Oocyte mitochondria are critical cellular organisms that regulate the potentiality of embryo development. Human and animal oocytes’ mitochondrial structure and function are reportedly seriously diminished following cryopreservation [108,109]. Kankanam Gamage et al. demonstrated how a supplementation of adipose stem cell mitochondria can positively affect the declined embryo development caused by cryopreservation-mediated cellular stresses and damages, and thus live birth rates [110]. 

Although the present review is mostly concentrated on female infertility, it is still worth mentioning that, as far as male fertility is concerned, spermatogenesis is known to be a gradual, orderly cascade process which comes to fruition through the precise regulation of genes, proteins, and various cytokines [111].

The protective effects of MSCs (human umbilical cord mesenchymal stem cells) are likely associated with their ability to secrete various cytokines which participate in testes development and hormone synthesis, improve spermatogenesis and the sperm maturation micro-environment, and affect sperm quality and male fertility [112]. Nagano M et al. provide a mechanism to evaluate the status of the stem cell population in selected infertile male patients that had shown how a xenogeneic transplantation of human germ cells using mice as recipients is feasible and could be used as a biological assay system to further characterize human spermatogonial stem cells [113]. Just as meaningful are the data reported by Văduva et al. [114], which point to cell-cloning technologies as an increasingly promising therapeutic avenue for the treatment of azoospermia, including the use of secondary spermatocytes, sperm cell cloning, and artificial sperm generation through the differentiation of stem cells and adult somatic cells into sperm cells. While still at the early stages based on animal models and despite a still-low level of efficiency, such techniques certainly hold great promise as treatment option for azoospermia-related infertility.

Chemotherapeutic drugs can cause reproductive damage due to their gonadotoxic effects on sperm quality and other aspects of male fertility. The study by Zhang Y et al. focuses on showing how stem cells can reportedly alleviate the damage caused by chemotherapy drugs and to play roles in reproductive protection and treatment [115]; this was conducted in order to investigate whether exosomes derived from human umbilical cord mesenchymal stem cells (hucMSC-derived exosomes) can repair injured endometrial epithelial cells (EECs) and reduce their death, and exhibit an anti-inflammatory effect against OGD/R (oxygen and glucose deprivation/reoxygenation) [116]. As reported in 2016 by multiple groups, scientists developed the ability to culture human embryos for 12 or 13 days [117], and in light of such developments, ethicists have called for the policy to be reconsidered, with some even suggesting that research should be allowed until the 21st or 28th day after fertilization [118]. Such a rule in fact risks being made obsolete by the apparently unstoppable progress in bioengineering. As thoroughly expounded upon by Anifandis et al. [119], three-dimensional embryo models have recently been generated through the in vitro mixing of embryonic and extra-embryonic stem cells via the identification and isolation of a human trophoblast stem cell population [119,120,121]. Another such avenue has led to the creation of expanded pluripotent stem cells (EPSCs) resembling epiblasts and hypoblasts among others [122], and yolk-sac-like cells (YSLCs) [123,124]. It is a widespread belief that gastrulation-like tissues will soon be generated, with animal models currently paving the way for such a progress to occur [125,126]. It is therefore a rather safe assumption that bioengineering, and its great potential, will soon yield totipotent synthetic embryos and beyond. Scholars and policy/law makers must be fully aware of the fact that innovations may outpace the ethics and legal precepts which guide us today. Such an evolution calls for a broad, concerted effort to update and adjust the standards and norms that aim to guarantee the ethical implementation of such techniques, whose growth is unstoppable and of huge benefit to countless patients [127]. Future prospects of such applications may greatly benefit, for instance, fertility preservation (FP), i.e., the maintenance of future reproductive capacity in cancer patients, especially of reproductive age, facing potentially gonadotoxic therapies [128] or surgical interventions affecting their reproductive capacity [129,130,131,132,133]. FP currently relies on oocyte cryopreservation or embryo-freezing through vitrification [134,135] as the most common approaches. Stress reduction through relaxation training or behavioral treatment has been demonstrated to improve conception rates, especially by virtue of the beneficial psychological support it can provide [136].

## 5. Conclusions

For infertile couples who cannot benefit from ART, stem-cells-based approaches can be a highly promising option, despite the lingering ethical quandaries and immunological uncertainties. More conclusive scientific data are still necessary for such techniques to be viable for mainstream use. The isolation of human ESCs (embryonic stem cells) is ethically controversial. Although ESCs are genetically unrelated to patients, their collection does entail the destruction of human embryonic tissue. Overall, stem cell research has brought about important new breakthroughs in the treatment of infertility. The common efforts towards untangling the complex web of ethical issues associated with this therapy need to be continued and expanded. International consensus will be vital in order to avoid a scenario in which citizens of countries where a given technique is illegal will have to travel to a country where it is not, which would discriminate against those who cannot afford such an option. The ultimate purpose is devising a well-balanced set of guidelines and evidence-based standards to harness the full potential of stem-cells-based therapeutic approaches, in an ethically and legally tenable fashion, for the sake of all those seeking to fulfill their reproductive potential.

## Figures and Tables

**Table 1:** Stem cells variants with potential uses in reproductive medicine.

Stem Cell Type	Distinctive Features	Reproductive Application
Embryonic stem cells (ESCs)	They are capable of self-renewal and can differentiate into different tissues (ectoderm, endoderm, mesoderm) [11]. They originate from blastocysts and express factors, Oct-4 [12]. Even if hES cells can give rise to all somatic tissues, they cannot form all of the other ‘extraembryonic’ needed for thorough development, e.g., the placenta and membranes; hence, they cannot form a whole new human being. Such features differentiate them from ‘totipotent’ fertilized oocyte and blastomere cells, which originate from the first cleavage divisions.	They can yield male and female gametes [13] through meiosis. ESCs play a key role in endometrial restoration [14]. However, to date, low levels of efficiency have been reported in terms of deriving germ cells from ESCs, with desired gamete function being observed in only one instance. Further research is needed on the derivative mechanism and the dynamics at the root of epigenetic signature establishment. Only primordial germ cells (PGCs) have so far been obtained in humans [14].
Induced pluripotent stem cells (iPSCs)	As described by Takahashi and Yamanaka [15] in 2006, such cells express different transcription factors such as Oct-4, klf 4, sox 2, c—myc. A 2023 study by Murakami et al. [16] based on animal models has achieved the generation of oocytes differentiated from iPSCs from male mice.	They originate from adult cells; thus, they are not as ethically controversial as ESCs. In addition, they are developed from the patient’s somatic cells, which avoids immune reaction [17,18], while the main drawback is genetic instability [19]. iPSCs share morphological similarities with ESCs, with the expression of ESC markers and telomerase activity, normal karyotypes, as well as the differentiation potential of the three main embryonic layers. Fundamentally, iPSCs are adult cells, genetically reprogrammed to resemble a given ESC through the expression of genes and other factors maintaining ESC characteristics. Though iPSCs are currently not yet ready to be transplanted, healthy autologous cells, i.e., immunocompetent ones for each individual patient, may one day be obtained through gene-editing technologies.
Mesenchymal stem cells (MSCs)	They have a plastic, adhesion quality, they express CD105, CD73, CD90 as markers, and they can give origin to osteoblasts, adipocytes, and chondroblasts [20,21]. The principal kinds of MSCs are documented by Saha et al. [11] in their review, and in great detail by Rizano et al. [22] in a broad-ranging 2023 review expounding upon their potential, as well as the pros and cons, in reproductive medicine: ✓ Bone marrow mesenchymal stem cells: they were studied by Owen et al. [23] for the first time in 1988. The injection of this kind of stem cell has been reported to improve endometrial thickness in a 2014 study by Jing et al. [24]. On the other hand, Wang et al. [25] used bone marrow mesenchymal stem cells to increase endometrial estrogen receptors in mice. ✓ Menstrual blood mesenchymal stem cells (hMensSCs): Liu et al. [26] demonstrated these cells improving ovarian function in mice, thanks to the transcription factor OCT-4. ✓ Endometrial stem cells (EnMSCs). ✓ Umbilical cord mesenchymal stem cells (UC-MSCs): easily obtainable, and with low immunological risk; can support ovarian function, reduce inflammatory cytokines, and improve fertility [27]. ✓ Amniotic fluid stem cells: thanks to VEGF, EGF, and BMP, they can increase ovarian function, preventing atresia [28]. ✓ Amnion-derived mesenchymal stem cells. ✓ Placenta-derived mesenchymal stem cells: they can improve folliculogenesis, thanks to the pathway PI3K/Akt [29]. ✓ Adipose-tissue-derived stem cells: in mice, they increase neovascularization and follicle proliferation [30].	These cells can be beneficial in ovarian and endometrial dysfunction by reaching ovarian tissue and restore its function via several cytokines and growth factors. MSCs are able to create new vessels and inhibit apoptosis and fibrosis [11]. Among these cells, fetal ones can reportedly rely on better telomerase activity and longer survival. They can be found in blood, bone marrow, liver, cordon blood, Wharton’s Jelly, amnion, and placenta [21]. Bone-marrow-derived stem cells can stimulate ovarian function, favorably affect ovarian and hormone levels, and possibly help to achieve pregnancy. In a 2017 research paper, Li et al. [31] reported that on days 14, 21, and 28 after transplantation of UC-MSCs into rats, a higher number of follicles was observed, the FSH levels had gone down, and the AMH and E2 levels had risen, all of which had a positive impact on ovarian reserve function. Transplantation of human BMSCs to mice can increase the ovarian weight, promote ovarian hormone production, and stimulate follicular development [32]. In addition, the transplantation of hMensSCs lead to higher ovarian weight, plasma E2 levels, and follicle numbers in mice [26,33]. HUMSCs have been shown in animal models to somewhat improve reproductive senescence through paracrine, anti-apoptotic, anti-fibrotic, angiogenic, anti-inflammatory immunomodulatory, and anti-oxidative stress effects [34]. Moreover, HUMSCs seem to be effective in terms of restored ovarian morphology and higher ovarian reserve capacity, and their potential in in vitro induction as germ cells also appears promising. Still, data from clinical trials are still inconclusive as to HUMSCs’ safety for the restoration of reproductive aging [34]. Amniotic fluid stem cells have shown an ability to differentiate into granulosa cells, which can counter or prevent follicular atresia and keep follicles healthy [35]. Still, more conclusive data are needed for human applications, since currently available results of MSC research in female-infertility-related diseases are mostly limited to findings in animal models and the underlying dynamics involving MSCs and several conditions causing female infertility have not yet been clarified [36].
Ovarian stem cells (OSCs)	They include pluripotent, very small embryonic-like stem cells (VSELs) and larger OSCs which are easily visualized in smears by scraping the ovarian surface. The potential of OSCs to differentiate into oocyte-like structures in vitro has been reported [37].	Johnson et al. [38] observed OSCs’ ability to induce follicle synthesis in animal models. In 2012, White et al. [39] used specific VASA markers to isolate ovarian stem cells from human ovarian cortex [24]. OSCs were even observed to foster ovarian regeneration and ovarian function overall [39]. Moreover, mitotically active germ cells from human ovaries, also known as germ stem cells (GSCs), can reportedly be purified and cultured in vitro to give rise to oocytes [39].
Spermatogonial stem cells (SSCs)	SSCs develop to form spermatozoa. During testicular homeostasis, SSCs self-renew to maintain the stem cell pool or differentiate to constitute a progeny of germ cells which sequentially transform into spermatozoa [40].	They play a key role in unlimited spermatogenesis in seminiferorous tubules [41]. SSCs are self-renewing cells and can produce a large number of committed progenitor cells, which in turn can differentiate into sperm. After being transplanted into the recipient’s testes, the sperm can be restored in a previously infertile patient [40]. Stem cells isolated from the testes of donor male mice were injected into the seminiferous tubules [42]. Donor spermatogonial stem cells can reportedly trigger spermatogenesis with normal morphological features in the testis, thus producing mature sperm. In humans, SSCs are responsible for the continuous production of male sperm [42,43].

**Table 2:** Legislative and regulatory state of affairs in six major European countries.

Country	Legislation Currently in Place	Relevant Legislative Provisions	Bioethics Oversight
Italy	Law 40, enacted on 24 February 2004, Regulation of Medically Assisted Human Reproduction [75].	The current legislative situation in the country is the outcome of a heated and drawn-out debate between supporters and opponents of embryonic stem cell research and ART. In 2005, the law was challenged in Italy’s highest court, the Constitutional Court, by opponents who included scientists seeking a review of the ban on the use of embryos for research. The court allowed a referendum on several parts of the law, including on whether or not the prohibition on embryo research could be relaxed. The referendum was held in 2005 but failed to reach the minimum 50% voter turnout. A 2009 Ministerial Decree that confined research funding to tissue (adult) stem cell research, so excluding embryonic stem cell research, has so far been unsuccessfully challenged by a number of Italian scientists following several appeal cases before the Italian courts.	The Italian National Ethics Committee instituted in 1990 to deal with the ethical legal and social implications linked to scientific research and technological applications on persons. The committee is made up of government-appointed scientists, physicians, and bioethicists. The committee has published many reports on embryo research and other related issues, but these have no binding authority. Other committees have recommended opposing opinions on some issues, including embryonic stem cell research [76].
France	Law on Bioethics, LOI n° 2011-814 [77]; French Public Health Code (article L1121-1) [78].	Research on human participants needs to meet specific standards (a protocol must be submitted in writing including the information document and the consent form). Specific criteria govern the collection of human material, including biobanking. According to article L1121-1 of the French Public Health Code, three research classes are deemed to involve human subjects: ✓ Interventional study (clinical trial). ✓ Interventional study (clinical trial) with minimal risk study. ✓ Non-interventional study (clinical trial) [78]. The 2011 law on bioethics, as amended in 2013, allows for research on human embryos and embryonic stem cells, provided that the following conditions are met: - Scientific relevance is acknowledged. - The research has a medical objective and cannot be conducted otherwise, i.e., without relying on human embryos or embryonic stem cells. - The research project meets the ethical standards for research on embryos and embryonic stem cells. - Embryos used for research must come from IVF, and no longer be part of a family project. Informed consent must be obtained from the donors’ couple, to be renewed after three months and revocable at any time.	Local Ethics Committee (“Comité de Protection des Personnes”) for ethical approval of the research project; French National Agency for the Safety of Medicines and Health Products (ANSM) for authorization of interventional studies and to be informed in case of other studies (interventional study with minimal risk and non-interventional study). French Ministry of Research and Health Regional Agency (“Agence Régionale de Santé”): The French Biomedicine Agency (“Agence de la Biomédicine”) authorizes research on human embryos and embryonic stem cells [77,78].
Germany	Embryo Protection Act (Embryonenschutzgesetz) 1991 [79]; 2002 Stem Cell Act (Stammzellgesetz) [80]; 2008 Act ensuring Protection of Embryos in connection with the importation and use of human embryonic stem cells [81].	Embryo research is heavily restricted in Germany: deriving embryonic stem cell lines is a crime. The German Constitution (Grundgesetz) itself enshrines embryo protection by stating that “human dignity is inviolable” and “everyone has the right to life and inviolability of his person.” At the same time, the freedom to pursue scientific research is also upheld. German law prioritizes adult stem cells for research under the 2002 Stem Cell Act (Stammzellgesetz) [80]. Embryonic stem cell lines can, however, be imported under strict conditions set by lawmakers. The 2002 Act set 1 January 2002 as ‘cut-off date’: imported ES cell lines must have been derived before that date, which was then was moved to 1 May 2007. In addition to these criteria, embryonic stem cell lines can only be used for research if they are vital in developing new medical and scientific knowledge.	The importation of stem cell lines for research must be approved by the Central Ethics Commission for Stem Cell Research (ZES), made up of scientists, physicians, and bioethicists. The German National Ethics Council (Geschäftsselle des Nationalen Ethikrat), instituted in 2007, provides guidance to policy and law makers and the public on scientific and medical issues that affect society and human health.
United Kingdom	Human Fertilisation and Embryology Act 1990, Schedule 2 [82]. Human Tissue Act 2004, Section 1 (9) [83]; Human Tissue (Quality and Safety for Human Application) Regulations 2007 [84].	Ethical approval is required for specific research projects. Human tissue held for a specific research project needs approval by a recognized Research Ethics Committee (REC) (or where approval is pending). Research on embryos and human embryonic stem cells is legal under the Human Fertilisation and Embryology Act 1990, Schedule 2 [82].	The ethical approval is delivered by a Research Ethics Committee (REC) and it must be applied for using the guidance provided by National Research Ethics Service (NRES) at the Health Research Authority. Tissue banks that have been approved by an REC can provide human tissues to researchers, who do not need to store them under a Human Tissue Authority license during the period of the research project, subject to certain requirements. The Human Fertilisation and Embryology Authority (HFEA) is in charge of regulating the storage of gametes and embryos and issuing licenses for research projects involving human embryos.
Spain	Law on Biomedical Research (Law 14/2007) [85]. Law 14/2006, of May 26 [85], on assisted reproduction techniques.	Spanish law expressly bans the creation of human pre-embryos (i.e., an embryo formed in vitro by a group of cells resulting from the progressive division of the egg cell, from the time it is fertilized until 14 days after) and embryos exclusively for experimentation purposes. In keeping with the gradualist perspective on the protection of human life outlined by Constitutional Court rulings 53/1985, 212/1996 and 116/1999. Still, techniques aimed at collecting embryonic stem cells for therapeutic or research purposes, without the creation of a pre-embryo or of an embryo exclusively for this purpose, are legal, in compliance with legislative standards. In addition, it is worth remarking that, although creating embryos for research is illegal, Law 14/2006 (which expressly forbids so-called reproductive human cloning) does allow supernumerary embryos to be donated for such purposes [86], for a specific research project or destroyed. Both options rest upon the informed consent of the embryo owner(s).	Guarantees Commission for the Donation and Use of Human Cells and Tissues, established under the Real Decreto 1527/2010 [87]. National Commission on Assisted Human Reproduction, established under Real Decreto 42/2010 [88].
Portugal	No specific legislation in Portugal currently governs stem cell research. Law n.º 32/2006, enacted on July 26, which regulates the use of medically assisted procreation [89], establishes the legal framework relative to quality and safety standards governing donation, collection, analysis, processing, preservation, storage, distribution, and application of human tissues and cells [90]; Law No. 21/2014, of April 16 (Clinical Investigation Law) [91].	The creation of embryos through MAP for research purposes is banned. Still, the scientific investigation of embryos for prevention, diagnosis, or therapeutic purposes, or to improve MAP procedures, is allowed under supervision. Legally usable embryos are: ✓ Cryopreserved, surplus embryos not part of a parental project (depends on prior, express, informed, and conscious consent of the intended beneficiaries); ✓ Embryos not viable for transfer or cryopreservation; ✓ Embryos with major genetic abnormalities, in the case of pre-implantation genetic diagnosis (on informed consent of those for which they were intended); ✓ Embryos obtained without fertilization by spermatozoa.	The use of embryos for scientific research purposes, limited to embryos produced for other purposes, always depends on the authorization of the experimentation by the National Council for Medically Assisted Procreation (CNPMA), established by Law 32/2006, of 26 July [89], which is charged with passing judgement on the ethical, social, and legal issues of medically assisted procreation.

## Data Availability

All data are available upon request from the corresponding author.
